# LC–MS peak assignment based on unanimous selection by six machine learning algorithms

**DOI:** 10.1038/s41598-021-02899-4

**Published:** 2021-12-03

**Authors:** Hiroaki Ito, Takashi Matsui, Ryo Konno, Makoto Itakura, Yoshio Kodera

**Affiliations:** 1 Department of Physics, School of Science, Kitasato University, 1-15-1 Kitasato, Minami-ku, Sagamihara, Kanagawa 252-0373 Japan; 2 Center for Disease Proteomics, School of Science, Kitasato University, 1-15-1 Kitasato, Minami-ku, Sagamihara, 252-0373 Japan; 3 Department of Biochemistry, School of Medicine, Kitasato University, 1-15-1 Kitasato, Minami-ku , Sagamihara, 252-0373 Japan

**Keywords:** Proteomic analysis, Mass spectrometry

## Abstract

Recent mass spectrometry (MS)-based techniques enable deep proteome coverage with relative quantitative analysis, resulting in increased identification of very weak signals accompanied by increased data size of liquid chromatography (LC)–MS/MS spectra. However, the identification of weak signals using an assignment strategy with poorer performance results in imperfect quantification with misidentification of peaks and ratio distortions. Manually annotating a large number of signals within a very large dataset is not a realistic approach. In this study, therefore, we utilized machine learning algorithms to successfully extract a higher number of peptide peaks with high accuracy and precision. Our strategy evaluated each peak identified using six different algorithms; peptide peaks identified by all six algorithms (i.e., unanimously selected) were subsequently assigned as true peaks, which resulted in a reduction in the false-positive rate. Hence, exact and highly quantitative peptide peaks were obtained, providing better performance than obtained applying the conventional criteria or using a single machine learning algorithm.

## Introduction

Liquid chromatography–mass spectrometry (LC–MS) has advanced remarkably in recent years, and LC–MS-based shotgun proteomics techniques enable the comprehensive identification and quantification of tryptic peptides. Further developments in high-resolution MS capabilities have enabled MS1-based quantitative comparisons of objective and control peptides from extracted ion chromatograms (XICs)^1, 2^. Shotgun proteomics techniques are thus commonly used in biological research (e.g., identification of disease-specific biomarkers)^3–5^. Although the isotope dot product (idotP) and mass error (∆M), calculated from MS1 spectra using Skyline^6, 7^, were adopted as comparative quantification criteria for peptide pairs in some other studies^8–12^, these conventional criteria are not sufficient to distinguish peptide peaks from noise. As such, the presence of noise peaks becomes a greater problem as the size of the dataset increases. Consequently, validating all extracted peaks requires manual inspection to eliminate noise peaks in the dataset.

Some recent investigations have used machine learning techniques to identify peptide peaks from large datasets and classify proteins in comparative analyses^13–20^. A mass precision algorithm was developed to extract the signal from the noise, thus improving quantitation using a random forest (RF) classifier and heuristic score^13^. Another algorithm has been released that identifies quantitative peaks from interfering peaks or poor chromatograms in targeted proteomics using a supervised machine learning approach^14^. Supervised machine learning approaches developed using quantitative results annotated by experts enable beginners to easily extract quantitative peak pairs with high accuracy. In contrast to the above advantage, however, false-positive and false-negative results can occur even when using datasets classified using supervised machine learning; consequently, false-positive peaks may reduce accuracy and introduce ratio distortion.

In this study, we adopted idotP and ∆M in addition to seven other informative features of chromatographic peaks. We examined these features using six different types of supervised machine learning algorithms to individually extract the peptide peaks. Our strategy evaluated each peak identified using six different algorithms; peptide peaks identified by all six algorithms (i.e., unanimously selected) were subsequently assigned as true peaks. Because unanimous agreement between all six algorithms leads to a reduction in the false-positive rate, the advantage of this system is that it enables extraction of more-exact and highly quantitative peptide peaks in comparison with a single supervised machine learning procedure or applying conventional criteria. Here, we report an example of such quantitative comparisons using our unanimous peak assignment procedure.

## Results and discussion

### Evaluation of training example

Tryptic peptides equivalent to 0.1 µg of homogenized total protein were analyzed using nanoLC-MS/MS. Peptide identification using Proteome Discoverer 1.4 yielded 5842 peptide fragments derived from 1214 proteins. The training example contained 380 peptides and 357 noise peaks. A total of 380 peaks with idotP ≥ 0.85 and |∆M| ≤ 10.0 ppm were manually annotated as peptide peaks (Supplementary Fig. S1A–S1D). Randomly selected signals were evaluated manually, resulting in 357 peaks, including peaks that fell within the criteria, which were assigned as the noise peaks (Supplementary Fig. S1E–S1H). The distributions in the ranges of features such as *m/z*, retention time, and intensity of all annotated peaks are summarized in Supplementary Fig. S2, and the data suggest that there was no bias between peptide and noise peaks, except with regard to peak intensity.

**Figure 1:**
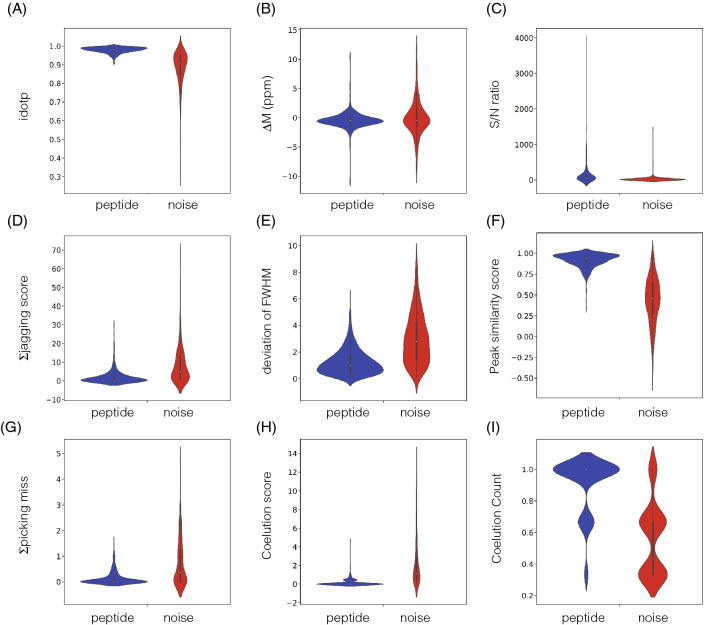
Violin plots of chromatographic features. (**A**) idotP, (**B**) average mass error among isotopes, (**C**) signal to noise ratio, (**D**) jagging score, (**E**) deviation of FWHM values among isotopes, (**F**) shape similarity score, (**G**) intensities at integral boundaries, (**H**) co-elution score, (**I**) co-elution count. Peptide and noise peaks are depicted in blue and red, respectively.

**Figure 2:**
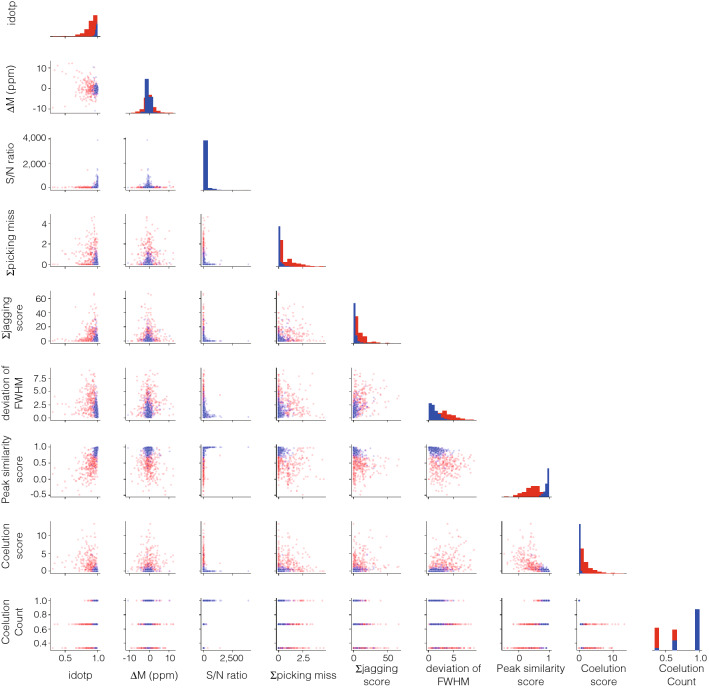
Correlations between chromatographic features. Correlations between combinations of two features among nine total features are shown. Peptide and noise peaks are depicted in blue and red, respectively.

The distributions of nine features between the 380 peptide peaks and 357 noise peaks were confirmed using violin plots (Fig. 1). Descriptions of the nine informative features are noted in Supplementary Table S1. With regard to idotP, the median value in the noise peaks was 0.90, and as a result, over half of the noise peaks were present in the extracted dataset set at a threshold of 0.85. Furthermore, the distributions of average mass error, jagging score, and standard deviation of full-width half maximum (FWHM) of the peptide peaks overlapped well with those of the noise peaks. These plots suggested that no parameter markedly distinguished the peptide and noise peaks. The peak distributions were also projected in two-dimensional plots generated from two of the nine features (Fig. 2). These two-dimensional plots did not enable the discrimination of peptide peaks from the dataset.

The first principal component (PC1) in the principal component analysis (PCA) explained most of the variation in the original variable features and correlated with the idotP variable in the initial space with an eigenvalue of 0.93. The eigenvectors of PC2 and PC3 represented the peak shape similarities among isotope peaks. The eigenvector of PC4 was also related to the peak shape similarities and peak co-elution scores. The cumulative contribution ratio from PC1 to PC4 was approximately 1.0. Therefore, all peaks shown in the nine features in the original space were represented in the degenerated space spanned by the four eigenvectors in the PCA space. The peak distributions in the three-dimensional projections of PC1–PC2–PC3, PC1–PC2–PC4, and PC2–PC3–PC4 are displayed in Supplementary Fig. S3. These projections revealed no threshold for the separation between peptide and noise peaks in the PCA space.

### Building and evaluating the machine learning algorithms

We concluded that no suitable threshold was found in the dataset subjected to PCA and therefore applied six machine learning algorithms, which are used for and are familiar to researchers in the proteomics field^13–20^, to classify the peptide peaks from the dataset. A total of 737 peaks, including 380 peptide and 357 noise peaks, were divided into a training set (418 data peaks; 219 peptide and 199 noise peaks) and a test set (319 data peaks; 161 peptide and 158 noise peaks) and then independently analyzed with the six different supervised machine learning algorithms using the dataset. Analysis of the learning curves for the six individual machine learning algorithms revealed that both the training and the cross-validation scores converged to a value > 0.85 (Supplementary Fig. S4). Increasing the number of peaks to 100 using RF^21^, extreme gradient boosting (XGB)^22^, k-nearest neighbor (KNN)^23^, linear support vector machine (SVM)^24^, artificial neural network (ANN)^25^ and Gaussian naïve Bayes (GNB)^26^ improved the training of the machine learning algorithms. Thus, the size of the training dataset was sufficient to build the machine learning algorithms without introducing overfitting problems. The permutation test (Supplementary Fig. S5) also suggested there was no significant overfitting with any of the machines.

Using the test set, the peak labels generated by the six machine learning algorithms were compared with the manually annotated peak assignments (Table 1). The GNB evaluation exhibited the lowest accuracy (89%), determined by dividing the number of correct predictions by the total number of peaks. In contrast to GNB, the ANN and XGB exhibited the highest accuracy (95%), and the ANN predicted 154 true peptide peaks and 150 true noise peaks in the test dataset. The precision, defined as the number of true peptides among all peptides predicted as true, indicated that the SVM, RF, XGB, and ANN achieved a high precision (approximately 96%). However, the KNN and GNB exhibited relatively poor precision, with 9% and 12.5% false-positive rates, respectively. The precision determined using the conventional criteria, which was assigned by idotP and ∆M, classified 35% of false peaks as peptide peaks. Although the machine learning algorithms were better classification tools in terms of identifying peptides as true peaks, nearly 4% of false positives derived from the machine learning algorithms may lead to inaccurate quantitative results. Therefore, in this study, peptide peaks unanimously selected as true peaks by the six machine learning algorithms were assigned as peptide peaks. Approximately 98.6% of identified peaks were consistent with the manually annotated peaks, and this unanimity enabled us to reduce the number of false positives to the lowest possible limit (Table 1). Although the total number of identified peptides was reduced slightly using our strategy rather than a single machine learning algorithm or previous criteria, our strategy for peak identification exhibited quite high fidelity.

**Table 1:** Confusion matrices used in this study. Precision rate was defined as the number of true peptides divided by the number of peptides predicted as true.

	Predicted peptides		Predicted as noise	
	True peptide	False-positive	False-negative	True noise
	Precision rate (%)			
Manual annotation	161	0	0	158
idotP and ∆M	160	87	1	71
	64.8%			
SVM	151	7	10	151
	95.6%			
RF	151	6	10	152
	96.2%			
XGB	151	5	10	153
	96.8%			
ANN	154	8	7	150
	95.1%			
KNN	152	15	9	143
	91.0%			
GNB	147	21	14	137
	87.5%			
Unanimous selection	140	2	21	156
	98.6%			

### Quantification analysis based on unanimous predictions

**Figure 3:**
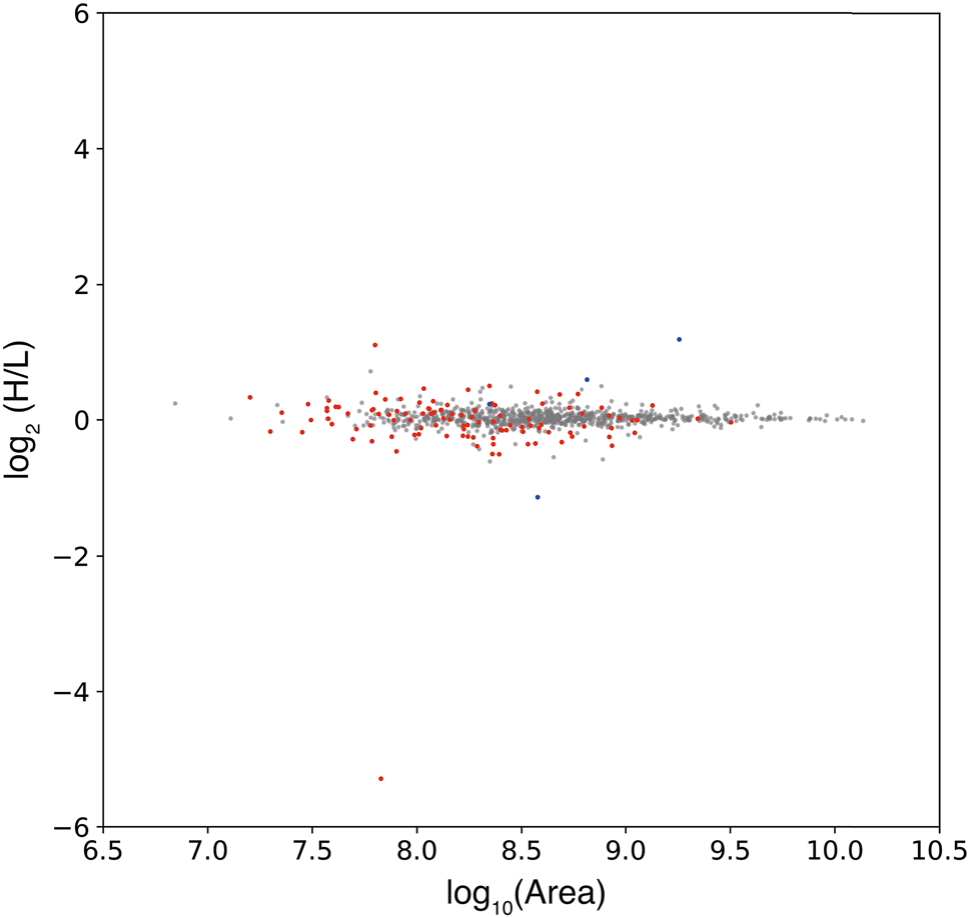
Quantitative analysis using the previous and current strategies. MA plot of peptide pairs extracted using the previous criteria and selected unanimously as true is shown. Peptides only selected unanimously and those classified only using the previous criteria are depicted in blue and red, respectively. Peptides identified by both are shown in grey. Equivalent amounts of light- and heavy-labeled proteins were mixed and then analyzed using LC–MS/MS. Thus, in principle, the peak area of light-labeled peptide would be consistent with the corresponding heavy-labeled peptide, and the log ratio would return to zero.

**Figure 4:**
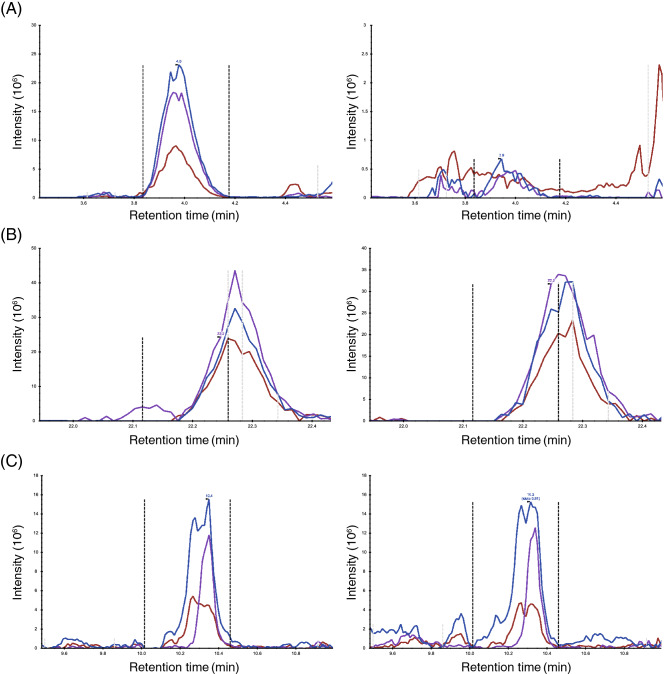
Profiles of chromatographic peaks. The chromatograms of M, M + 1, and M + 2 in heavy- (left) and light-labeled (right) samples are depicted in blue, purple, and brown, respectively. (**A**–**C**) Examples of outliners in the unanimous selection. (**A**) Case of a peak with a low signal intensity misidentified as a true peak. Sequence, K[+ 25]SAPATGGVK[+ 28]K[28 +]PHR; charge = + 4, (**B**) Case of an incorrect integral of interval. Sequence, DGK[+ 28]YHSIK[+ 28]EVATSVQLTLR; charge = + 3, (**C**) Case of differing shapes among isotopes. Sequence, LK[+ 28]QLAAEQGK[+ 28]DIR; charge = + 4.

The unanimous prediction strategy was applied to the analysis of a mixture of equivalent quantities of proteins labeled with light and heavy dimethylations as an example for quantitative comparisons. The peptide identification workflows using this and previous approaches are indicated in Supplementary Fig. S6. In the previous approach, peptide peaks were identified based on the following criteria: idotP ≥ 0.9 and |∆M| ≤ 6 ppm, resulting in the prediction of 939 peak pairs. Using the unanimous selection approach, a total of 893 peak pairs were identified (Supplementary Table S2). The distributions of retention time, intensity, and *m/z* for these peak pairs are shown in Supplementary Fig. S7. No deviation in retention time was observed using both approaches. The peak intensity frequency extracted by the approach converged to 1 × 10^8^ with a Gaussian-like distribution. Analysis of the variations in *m/z* showed that an increase in *m/z* was related to a decrease in the number of identified peaks. Although the unanimous algorithm prediction approach identified fewer peaks than were identified using the previous criteria, no significant distribution differences between the approaches were found. Therefore, the unanimous algorithm prediction approach identifies peptide peaks from a search space similar to that annotated using our previous criteria. Log ratio-mean average (MA) scattering plots were generated to visualize the distributions of peak pairs extracted using both approaches (Fig. 3). The average of the log-ratios of the unanimous algorithm prediction approach was 0.030, with a deviation of 0.012. In contrast, that of the former criteria yielded a slightly lower value of 0.021 but a higher deviation value of 0.022. In the MA plots, the peptides assigned solely by the former criteria appeared within a broad range of intensity, with an inaccurate ratio (Fig. 3). Focusing on pairs solely assigned by the former criteria, some of the peaks were assigned as peptides by the former criteria but confirmed as noise peaks by visual analysis (Fig. 4A). Some peaks were located out of the interval of the integral (Fig. 4B). The chromatogram of the isotopes exhibited different shapes from chromatograms of other isotopes (Fig. 4C). In contrast to the previous approach, the unanimous algorithm prediction approach produced fewer peptide peaks with inaccurate ratios (Fig. 3). Convergence of deviations was observed, suggesting that the unanimous algorithm prediction approach is useful for comparative quantification of peptides with high accuracy.

## Conclusion

Shotgun proteomics using high-resolution MS enables us to conduct MS1-based quantitative comparisons of objective and control peptides from the XIC^1, 2^. To identify the proteins involved in physiologic and/or pathologic processes based on abundance using shotgun proteomics, poor chromatographic peaks must be excluded from complex LC–MS/MS spectra when using conventional criteria, such as idotP and ∆M^8–12^, and then quantifications based on the areas of extracted peaks of identified proteins can be compared. Recently, deep proteomic techniques have been developed that are capable of detecting weak peptide signals, thus introducing the problem of determining how to treat these weak signals for deeper quantification of protein/peptide abundance. However, manually annotating a large number of signals from a vast dataset is impractical, and it is difficult for an investigator to maintain consistent application of judgment criteria during peak annotation. In this study, we introduced six machine learning algorithms to successfully extract a higher number of peptide peaks with high accuracy and precision. Although machine learning algorithms are reliable classification tools for identifying peaks as true, single machine learning algorithms are associated with false-positive rates of at least 5%, which can lead to inaccurate quantitative results due to ratio distortion. In contrast to use of a single machine learning algorithm, our strategy evaluated each peak identified by six different algorithms, and those peaks selected unanimously by the algorithms as true were assigned as peptide peaks, resulting in a reduction in the false-positive rate to 1.4%. Although the high positive rate of unanimous selection is at the cost of the false-negative rate, the advantage of this strategy is that unanimous selection reduces the rate of false positives. The important point regarding this strategy is that reducing the number of false-positive elements, which pollute the true set, makes more-exact and highly quantitative peptide peak identification possible compared with use of a single machine learning algorithm or application of conventional criteria. Furthermore, our strategy also recorded how many machine learning algorithm selections were true. Consequently, we could identify an obscure peak with the score calculated from the number of selections returned to 0, 0.17, 0.33, 0.5, 0.66, 0.83, to 1, enabling re-assignment of the peak as an object peak.

Recent data-independent acquisition (DIA) MS-based techniques enable deep proteome coverage with relative quantitative analysis^27^, resulting in an increase in the identification of very weak signals from very large LC–MS/MS spectral datasets. The identification of weak signals using an assignment strategy with poorer performance resulted in inaccurate quantification and misidentification of peaks, along with ratio distortion. In this study, we developed a new peak assignment strategy based on unanimous selection by multiple machine learning algorithms to enable highly sensitive peak annotation results with a significantly lower false-positive rate. When coupled with DIA techniques, this strategy could enable determination of trace amount differences in protein abundance in cells and/or tissues, thereby providing new insights into physiologic and pathologic mechanisms in the near future.

## Materials and methods

### Sample preparation

A C57BL/6 adult male mouse was purchased from CLEA Japan, Inc. (Tokyo, Japan). The whole liver of single mouse was homogenized on ice using a BioMasher II (Nippi, Tokyo, Japan) for 3 min with 1 mL of phase-transfer surfactant [PTS; 12 mM sodium deoxycholate, 12 mM sodium *N*-lauroylsarcosinate, and 200 mM triethylammonium bicarbonate (TEAB)]^28^. Aliquots of the homogenate were sonicated in a Bioruptor sonicator (SONIC Bio Co., Kanagawa, Japan) for 30 min (30 s on/30 s off, high setting) while on ice water. Insoluble materials were removed by centrifugation at 19,000*g* for 15 min at 4 °C. The protein concentration was measured using a NanoDrop spectrophotometer (Thermo Fisher Scientific, Waltham, MA, USA) and adjusted to 1 µg/µL with PTS. Protein extraction samples were flash-frozen using liquid nitrogen and then stored at − 80 °C until use.

For evaluation of the machine learning algorithms, proteins extracted from 20 µg of mouse liver were resuspended in 20 µL of PTS and incubated with the addition of 2 µL of 200 mM Bond-Breaker TCEP solution (Thermo Fisher Scientific) for 30 min at 50 °C to cleave the disulfide bonds, and then the solution was further incubated on ice for 10 min. The reduced proteins were then alkylated with 2 µL of 375 mM iodoacetamide and 200 mM TEAB in the dark at room temperature for 30 min. The alkylation reaction was quenched by addition of 2 µL of 400 mM l-cysteine and incubation in the dark for 10 min at room temperature. The sample was digested with 200 ng each of trypsin and lysyl endopeptidase for 18 h at 37 °C. The reaction mixture was then mixed with a 1.5 × volume of 1.7% trifluoroacetic acid (TFA) and subsequently centrifuged at 19,000*g* for 15 min at 4 °C. The supernatant was desalted using StageTips with a C18 Empore disk membrane, as described previously^29^. The fraction was eluted using 50% acetonitrile (ACN) and 0.1% TFA and then freeze-dried. The freeze-dried sample was resuspended with 20 µL of 3% ACN and 0.1% formic acid (FA) using a combination of vortexing and ultrasonic agitation in a Bioruptor sonicator (30 s on/30 s off, high setting) for 10 min each while on ice water. The sample was analyzed using a quadrupole Orbitrap benchtop mass spectrometer (Q-Exactive, Thermo Fisher Scientific) equipped with an EASY-nLC 1000 system (Thermo Fisher Scientific). Tryptic peptides were injected directly onto an analytical column (C18, particle diameter 3 µm, 0.075 mm × 125 mm; Nikkyo Technos, Japan). Tryptic peptides were separated with a gradient of solvents A (0.1% FA) and B (0.1% FA and 90% ACN) (0–1 min, 5–10% B; 1–20 min, 10–25% B; 20–26 min, 25–50% B; 26–27 min 50–80% B) at a flow rate of 300 nL/min using the EASY-nLC 1000. Peptides were introduced from the chromatography column to the Q-Exactive. Some parameters of the MS spectra were as described previously^9^. MS1 spectra were collected over the scan range 350–900 *m/z* at 70,000 resolution to hit an automatic gain control (AGC) target of 1 × 10^6^. The AGC target value for fragment spectra was set at 1 × 10^5^. The 20 most-intense ions with charge states of 2^+^ to 4^+^ that exceeded an intensity of 2.0 × 10^3^ were fragmented.

For quantitative comparisons, proteins extracted from 20 µg of mouse liver dissolved in 20 µL of PTS were dimethylated with 8 µL of 0.6 M NaBH_3_CN and 16 µL of 4% ^12^CH_2_O (light-labeled) or 4% ^13^CH_2_O (heavy-labeled) for 10 min at room temperature. The dimethylation reaction was quenched by addition of 8 µL of 1% NH_3_ and incubation for 1 min, and then the light- and heavy-labeled samples were mixed. A total of 58 µL of the mixture sample was precipitated by the addition of 700 µL of ACN followed by the addition of 25 µL of 5% TFA. After centrifugation at 19,000*g* for 15 min at 4 °C, the supernatant was discarded to collect the precipitate. The precipitate was dissolved with 20 µL of PTS, and the subsequent procedures of alkylation, digestion, and LC–MS analysis were performed according to the above procedures described for evaluation of the machine learning algorithms.

Peptides were introduced to the Q-Exactive from an analytical column (C18, particle diameter 3 µm, 0.075 mm × 125 mm; Nikkyo Technos). Tryptic peptides were separated with a gradient of solvents A and B (0–29 min, 5–30% B; 29–37 min, 30–55% B; 37–38 min, 55–80% B) at a flow rate of 300 nL/min using the EASY-nLC 1000. MS1 spectra were collected over the scan range 350–1400 *m/z* at 140,000 resolution to hit an AGC target of 3 × 10^6^. The two most-intense ions with charge states of 2^+^ to 4^+^ that exceeded an intensity of 2.0 × 10^5^ were fragmented. Other parameters were set as described for evaluation of the machine learning algorithms.

All raw data files obtained in the LC–MS/MS analyses were deposited in the ProteomeXchange Consortium (http://proteomecentral.proteomexchange.org) via the jPOST partner repository (http://jpostdb.org)^30^ with the dataset identifiers PXD027824 for ProteomeXchange and JPST001287 for jPOST.

### Protein identification

LC–MS/MS data were searched against the mouse UniProt sequence database (release 2018; 25,131 entries, reviewed). Database searches were performed using the SEQUEST algorithm incorporated into Proteome Discoverer 1.4.0.288 software (Thermo Scientific) with the following parameters: enzyme, trypsin; maximum missed cleavage sites, 3 for evaluation of machine learning or 2 for quantitative comparisons; precursor mass tolerance, 6 ppm; fragment mass tolerance, 0.02 Da; fixed modification, cysteine carbamidomethylation; variable modification, methionine oxidation. For quantitative comparisons, light-labeled dimethylation (+ 28 Da) at lysine and heavy-isotope labeled dimethylation (+ 34 Da) at lysine were adapted as the search parameters. Peptide identification was filtered to a false discovery rate (FDR) of < 1%.

XICs for precursor ions were obtained using Skyline 20.1.0 (http://proteome.gs.washington.edu/software/skyline)^6, 7^ based on the identified peptide library. The spectrum library was imported from the msf file generated by Proteome Discoverer with a cutoff score of FDR = 0.99. Peptide settings were as follows: enzyme, trypsin KR/P; maximum missed cleavages, 2; minimal length of peptide, 7; maximal length, 30; modifications, carbamidomethyl (Cys), oxidation (Met); maximum variable mods, 5. Transition settings were as follows: precursor charges, 2^+^–4^+^; type, p (precursor); ion mass tolerance, 0.02 *m*/*z*; isotope peaks included, count 3; mass analyzer, Orbitrap; resolution, 70,000 at 200 *m*/*z*; use only scans within 5 min of predicted retention time; isotope labeling enrichment, default.

### Extraction of informative features from chromatographic peaks

Nine types of informative features of the chromatographic peaks were extracted using Skyline: idotP, average mass error, signal-to-noise ratio, standard deviation of the intensity of FWHM of isotope peaks, average retention time, intensity at chromatographic peak boundary, shape similarity, and co-elution score (Supplementary Table S1 and Supplementary Fig. S8). Jagging score was defined as the number of data points lower than the FWHM within an integral interval of the peak. Shape similarity score was defined as the Pearson product-moment correlation coefficient generated based on the similarity in shapes of chromatographic peaks of isotopes. The co-elution score was defined as the average shift in the cross-correlation function for each pair of isotopic peak traces within the window of the selected peak, as described in a previous report^31^. For all features, missing values were replaced with a zero.

### Peak extraction and assignment

All values in each feature parameter were scaled using min–max normalization. Subsequently, the dimensionality from the original feature space was reduced using PCA. We selected PCA components as inputs and then applied them to the SVM^24^, ANN^25^, KNN^23^, and GNB^26^ algorithms. The values of all feature parameters not subjected to min–max normalization and PCA were entered into other machine learning algorithms, RF^21^ and XGB^22^. The *k*-fold cross-validation (*k* = 5) approach was used to avoid the overfitting problem, and the hyper-parameters were optimized as described previously^32^. Optimization of hyper-parameters were applied for 5 machine learning algorithms, except GNB. True peaks and noise peaks in the training example were annotated manually.

### Quantification

Peptide pairs for which both the light- and heavy-labeled peptides were identified were chosen for comparative quantification. The sum of the XIC area of three ion precursors (monoisotopic mass [M] and isotopic masses [M + 1 and M + 2]) generated from the respective peptides was determined as the corresponding peak area.

### Coding environment

Python 3.7.7 was used to perform the machine learning analyses using the following imported libraries; numpy 1.19.1, pandas 1.1.0, scikit-learn 0.23.1, xgboost 0.9, matplotlib 3.2.2, and seaborn 0.10.1. Figures were prepared using matplotlib and seaborn. FeatureExtract.py and MachineLearning.py were used for extraction of chromatographic features and execution of the machine learning algorithms, respectively. Both python scripts are shown in the Supplementary Materials. The sample data, training data and report template for skyline are also included in the Supplementary Materials.

### Ethics approval

All the animal experimental procedures were approved by the Animal Experimentation and Ethics Committee of Kitasato University School of Medicine (permission number: 2020051). Procedures were performed in compliance with the ARRIVE guidelines and with guidelines for animal experiments by Kitasato University School of Medicine.

## Supplementary Information

Supplementary Information 1.

Supplementary Information 2.

Supplementary Information 3.

Supplementary Information 4.

Supplementary Information 5.

Supplementary Information 6.
