# Supplementary material for: LC–MS peak assignment based on unanimous selection by six machine learning algorithms
Source: Sci Rep. 2021 Dec 3;11:23411. doi: 10.1038/s41598-021-02899-4 (PMC8642397; doi:10.1038/s41598-021-02899-4)
Supplement: Supplementary file 1 — Supplementary Information 1. [file 41598_2021_2899_MOESM1_ESM.pdf]

Supporting Information for

LC-MS peak assignment based on unanimous selection by six machine learning algorithms

Hiroaki Ito[1, #], Takashi Matsui[1, 2, #], Ryo Konno[1], Makoto Itakura[2, 3] and Yoshio Kodera[1, 2, *]

[1] Department of Physics School of Science, Kitasato University, 252-0373 Japan.

[2] Center for Disease Proteomics, School of Science, Kitasato University, 252-0373 Japan.

[3] Department of Biochemistry, School of Medicine, Kitasato University, 252-0373 Japan.

[*]Correspondence: Prof. Yoshio Kodera. E-mail: kodera@kitasato-u.ac.jp.

Postal address: 1-15-1 Kitasato, Minami-ku, Sgamihara, Kanagawa 252-0373, Japan.

[#]Both authors contributed equally to this work.

Table of contents

Supplementary Tables and Figures

Table S1.	Description of informative features used in machine learning.
Table S2.	Number of peptide identifications and number of peptide pairs for the quantitative analysis.
Figure S1.	Profiles of chromatographic peaks in the training dataset.
Figure S2.	Distributions of peaks.
Figure S3.	Three-dimensional projections of the principal components.
Figure S4.	Learning curve.
Figure S5.	Permutation test.
Figure S6.	Workflow of conventional peak extraction and unanimous selection strategies.
Figure S7.	Distributions of quantitative peaks.
Figure S8.	Theory of co-elution score and co-elution count calculations.
Figure S9.	Receiver operating characteristic curves of the machine learning algorithms.

Supplementary scripts and example data

FeatureExtract.py	Extraction of chromatographic features from Skyline output.
MachineLearning.py	Execution of unanimous selection.
Skyline_OutPut_File.csv.zip	Sample data (Skyline output)
SkylineReportFile_Template.skyr	Report template for Skyline
TrainingDataSet.xlsx	Training set (manual assignment of 418 peaks)

Supplementary Table S1. Description of informative features used in machine learning.

Feature	Description
idotP	Isotope dot product calculated between theoretical and observed extracted ion currents.
ΔM	Mass error difference between theoretical and observed, and the average of mass error among isotopes.
SN ratio	Signal-to-noise ratio of monoisotopic mass (noise intensity defined as 1.0×10^6).
Σ jagging score	Sum of the number of data points lower than the full-width half-maximum (FWHM) within an integral interval of the peak.
Deviation of FWHM	Standard deviation of FWHMs among three isotopes.
Peak similarity score	Pearson product-moment correlation coefficient generated from similarity in shape between the chromatographic peaks of isotopes.
Σ PickMiss	Sum of intensities at chromatographic peak boundaries.
Co-elution score	Cross-correlation function of each isotope peak within the window of the selected peak calculated against each other. The

	co-elution score is the average shift in cross-correlation functions for the corresponding isotope peak as seen in mProphet [1].
Co-elution count	The co-elution count is the number of similar chromatographic peaks at the maximum value of the cross-correlation function within the windows of the selected peaks against each other divided by the number of corresponding isotopes (number of isotopes = 3). The cross-correlation matrix is the same as for the co-elution score.

Supplementary Table S2. Number of peptide identifications and number of peptide pairs for the quantitative analysis.

Category	No. of peptides
**Whole identified peptides**	4,331
**Identified peptides with dimethylated Lys**	1,145
**Peptides selected using multiple algorithm strategy**	893
**Peptides selected using previous criteria**	939

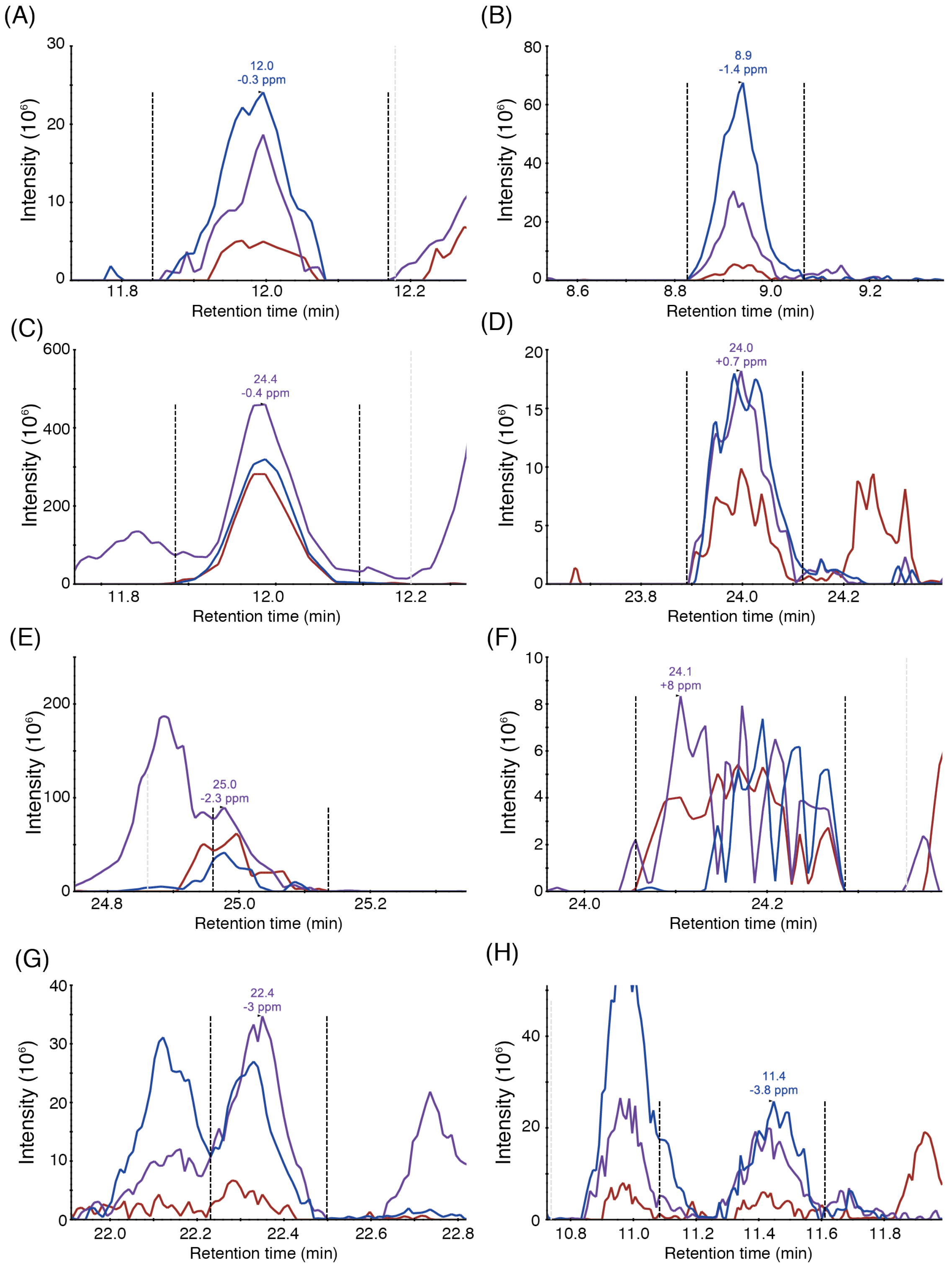

Supplementary Figure S1. Profiles of chromatographic peaks in the training dataset. (A-D) Four examples of manually annotated peptide peaks, sequences of (A)

RIPYTPGEIPK (charge = +3), (B) GYFEDR (charge = +2), (C) LTLYDIAHTPGVAADLSHIETR (charge = +4), and (D) ELAPYDENWFYTR (charge = +2). (E-H) Four examples of signals assigned as noise peaks. The chromatograms of M, M+1, and M+2 are depicted in blue, purple, and brown, respectively.

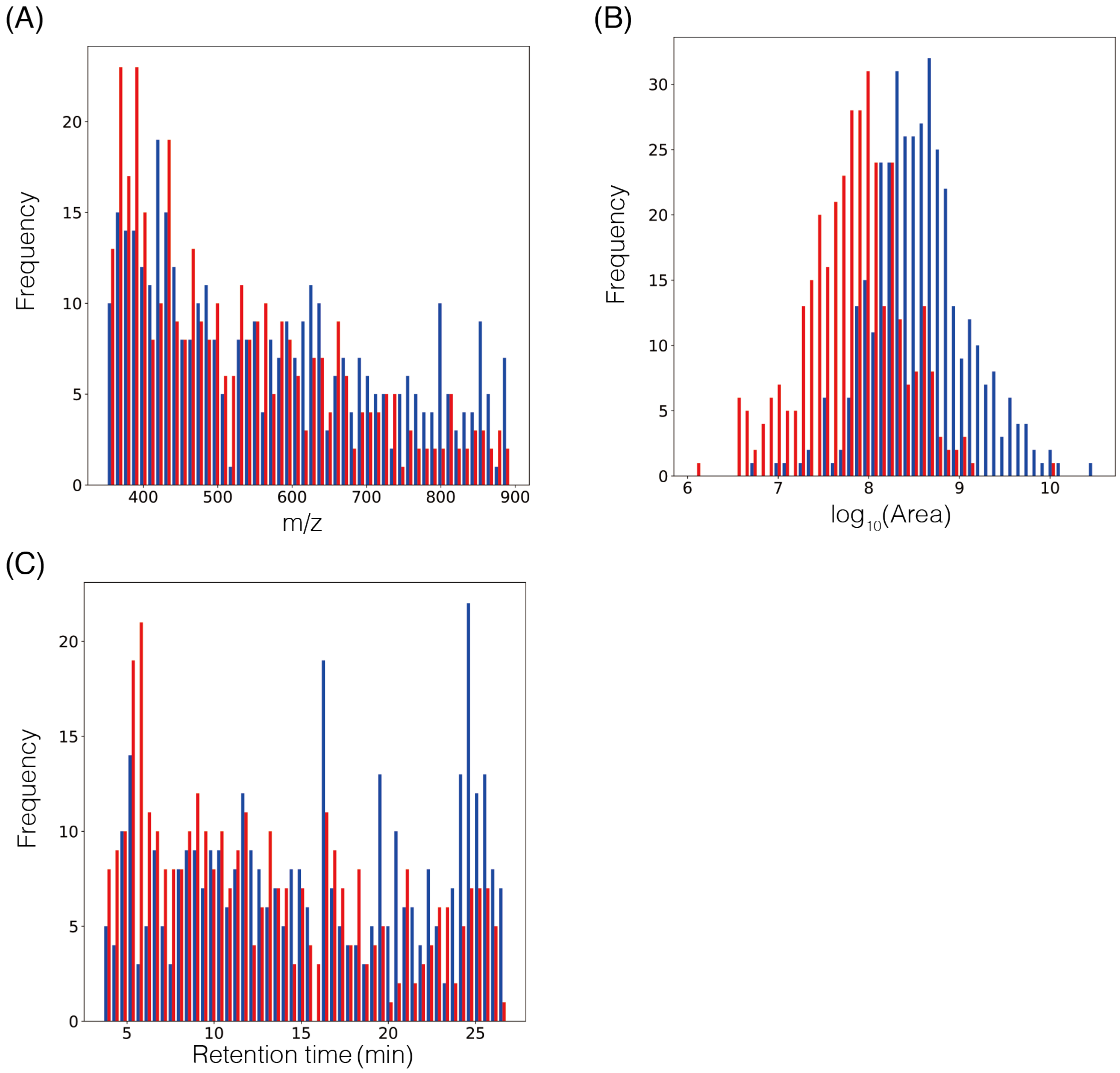

Supplementary Figure S2. Distributions of peaks. (A) Peak *m/z* range, (B) distribution of peak intensity, and (C) variation in retention time are shown. Peptide and noise peaks are depicted in blue and red, respectively.

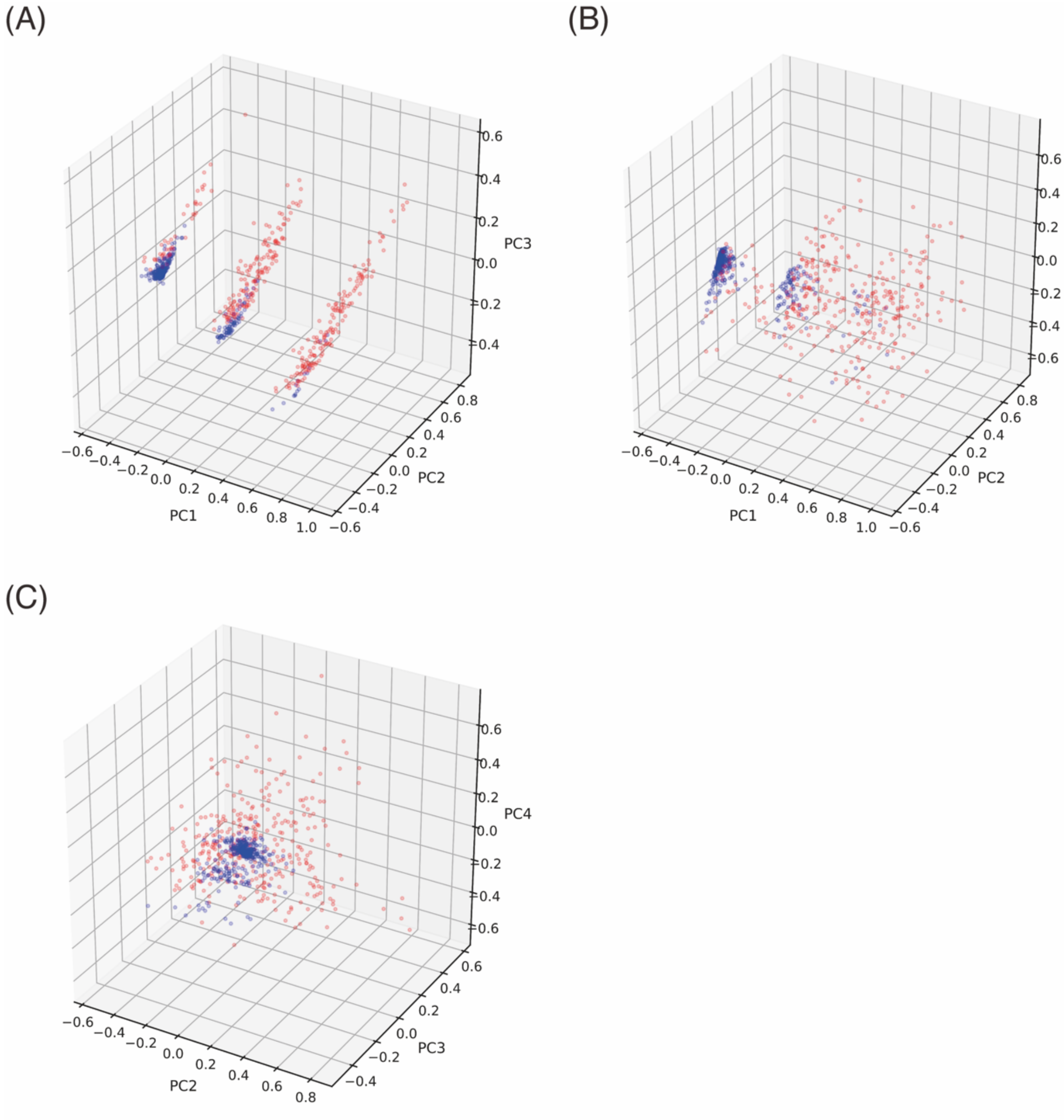

Supplementary Figure S3. Three-dimensional projections of the principal components. Projections spanned by (A) PC1-PC2-PC3, (B) PC1-PC2-PC4, and (C) PC2-PC3-PC4. Peptide and noise peaks in the training dataset are depicted in blue and red, respectively.

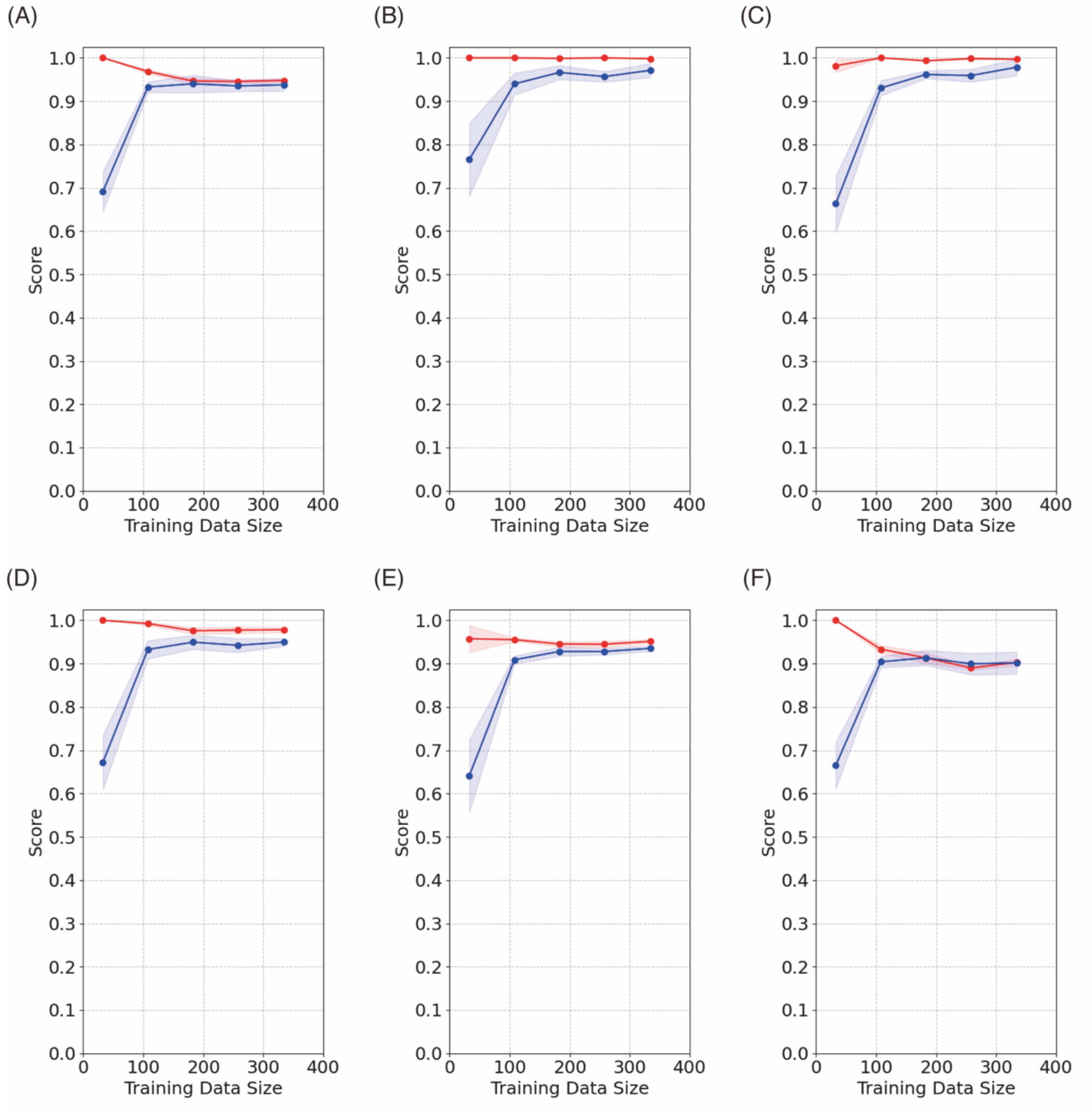

Supplementary Figure S4. Learning curves. Learning curves of (A) SVM, (B) RF, (C) XGB, (D) ANN, (E) KNN, and (F) GNB are shown. Training and cross-validation sets varying the size of the training set are depicted in red and blue, respectively. The *k*-fold cross-validation (k = 5) approach was used to divide the training and cross-validation sets from 418 data peaks. The size of the cross-validation set was one-fifth of the total 418 peaks. Deviation ranges of the training and cross-validation sets are shown in red

and blue, respectively.

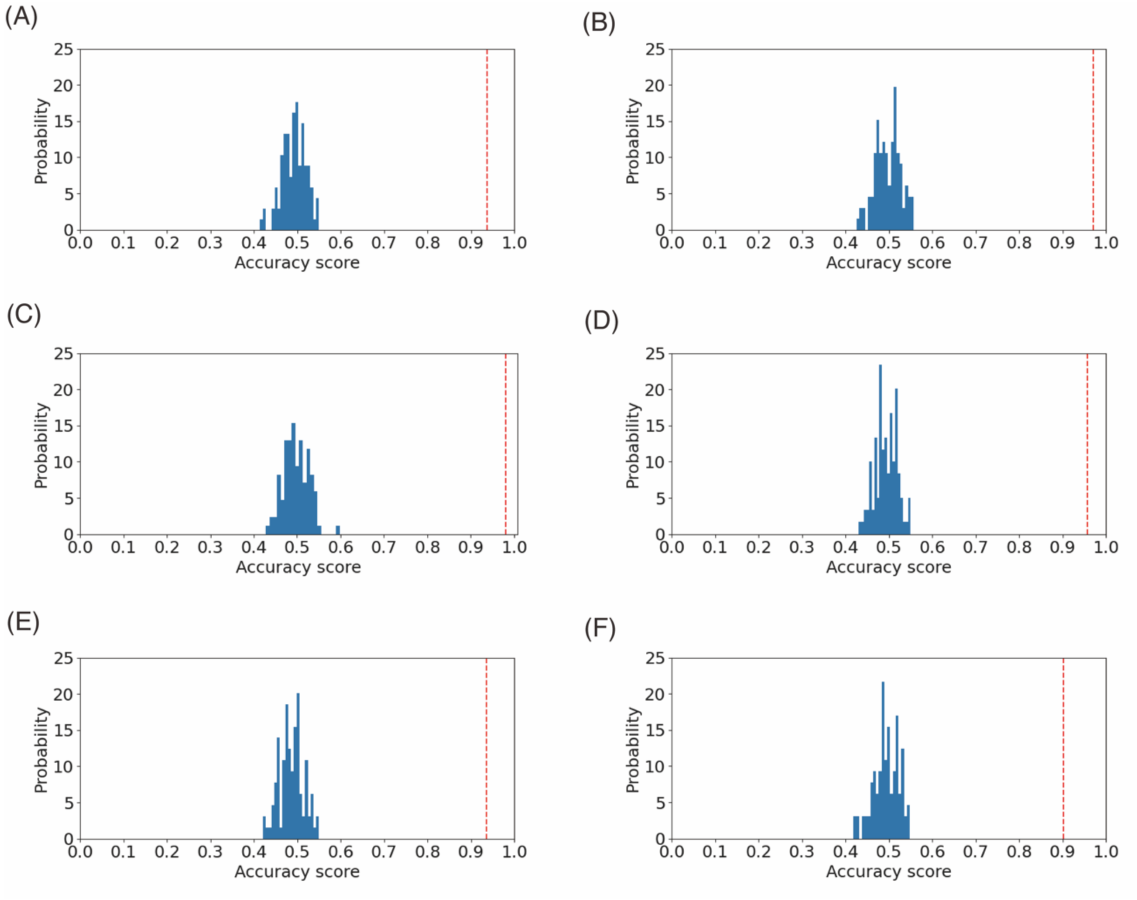

Supplementary Figure S5. Permutation test. Distribution of the scores of (A) SVM, (B) RF, (C) XGB, (D) ANN, (E) KNN, and (F) GNB are shown. The permutation test (*n* = 100) was applied to construct the random distribution of the training set with the *k*-fold cross-validation (k = 5). The distribution of the randomized data is depicted in blue. The score of the original data is depicted by the red dashed line. The random classifier converged well to 0.5.

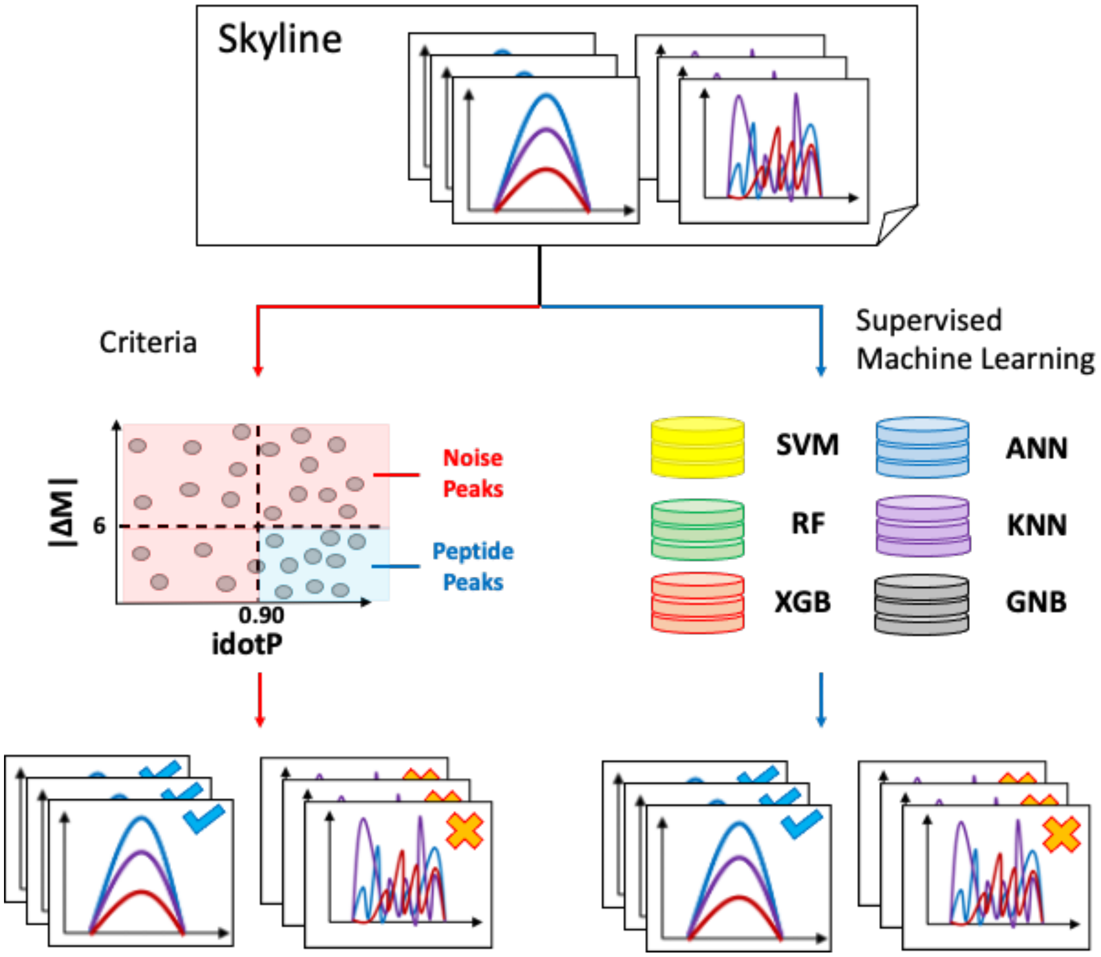

Supplementary Figure S6. Workflow of conventional peak extraction and unanimous selection strategies.

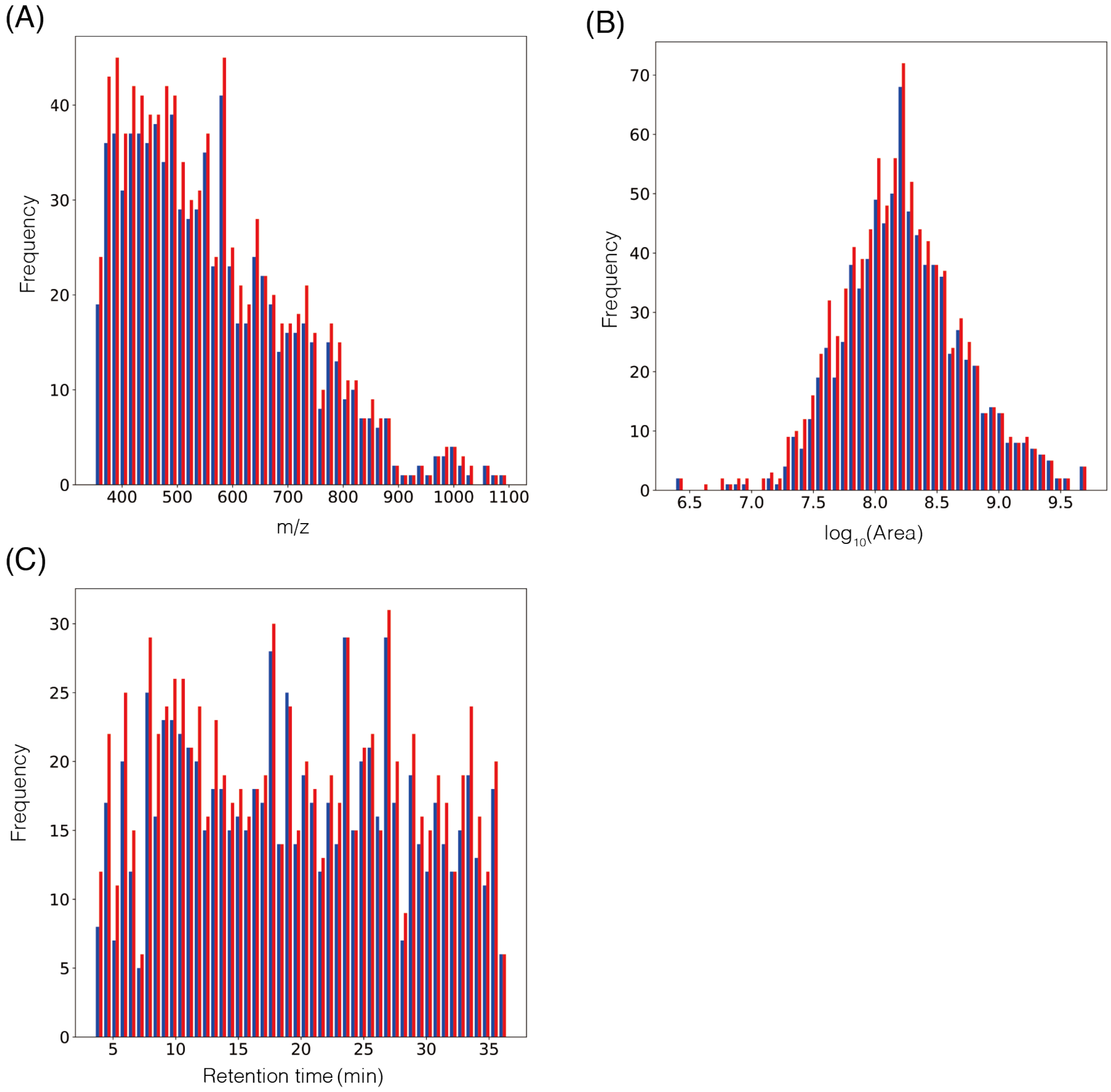

Supplementary Figure S7. Distributions of quantitative peaks. Frequencies of (A) *m/z*, (B) peak intensity, and (C) peak elution variation by retention time. Peptides selected unanimously and classified using previous criteria are depicted in blue and red, respectively.

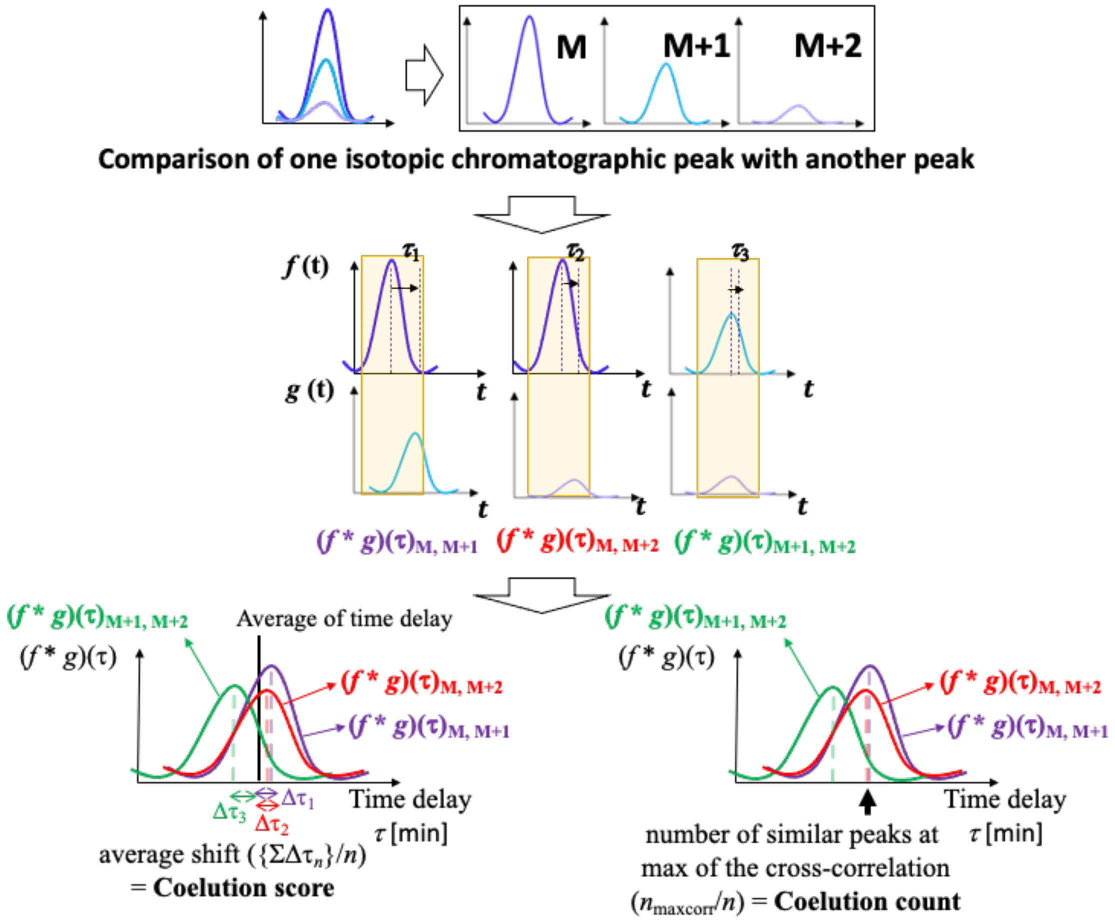

Supplementary Figure S8. Theory of co-elution score and co-elution count calculations. The co-elution score and count are measures of co-elution based on the shift in the cross-correlation function between each pair of isotopic peaks, as referenced from mProphet [1], with slight modifications. The 'n' term represents the number of measured isotopes (n = 3 in this study).

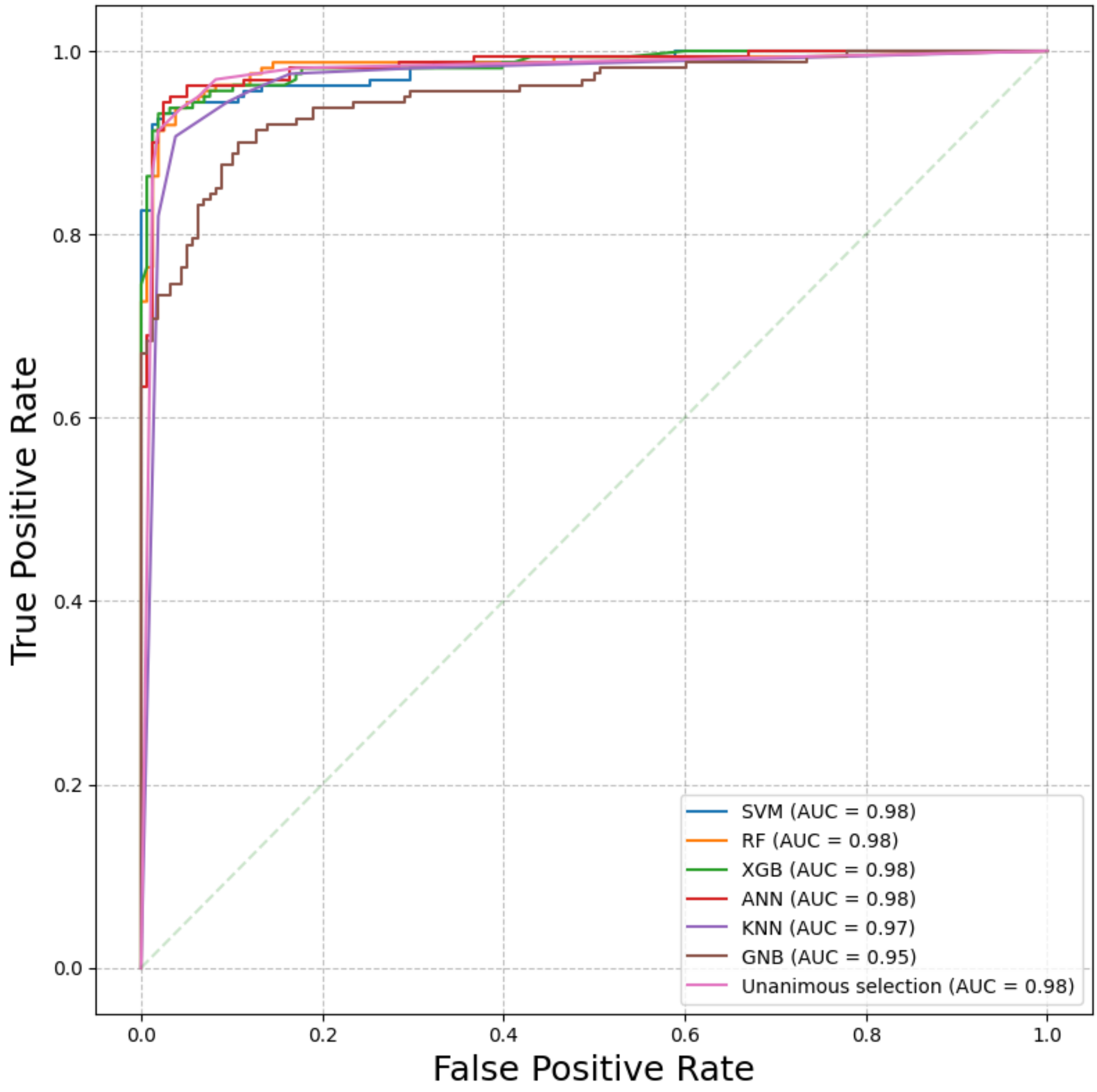

Supplementary Figure S9. Receiver operating characteristic curves of six individual machine learning algorithms. Area under the curve (AUC) for SVM, RF, XGB, ANN, KNN, GNB, and unanimous selection depicted in blue, orange, green, red, purple, brown, and pink, with values of 0.98, 0.98, 0.98, 0.98, 0.97, 0.95, and 0.98, respectively.

References

1. Reiter, L. *et al.* mProphet: automated data processing and statistical validation for large-scale SRM experiments. *Nature Methods* **8**, 430–435 (2011).
